# Global cell-by-cell evaluation of endothelial viability after two methods of graft preparation in Descemet membrane endothelial keratoplasty

**DOI:** 10.1136/bjophthalmol-2015-307534

**Published:** 2016-01-06

**Authors:** Maninder Bhogal, Maria S Balda, Karl Matter, Bruce D Allan

**Affiliations:** 1 Department of Corneal and External Disease, Moorfields Eye Hospital, London, UK; 2 Department of Cell Biology, University College London, Institute of Ophthalmology, London, UK

**Keywords:** Cornea, Eye (Tissue) Banking, Treatment Surgery

## Abstract

**Purpose:**

To describe a novel method of global cell viability assessment for Descemet membrane endothelial keratoplasty (DMEK) and the comparison of two contemporary methods of donor tissue preparation.

**Methods:**

DMEK transplants were prepared using two different methods: liquid bubble separation and manual peeling (n=8 each group). Samples were incubated with Hoechst, calcein-AM and ethidium homodimer prior to mounting on a curved imaging chamber. Z-stacked fluorescence microscopy images were combined to produce an in-focus global image capable of resolving all cell nuclei. Image processing software was used to define a calcein-positive live cell area, count all cell nuclei within this area and subtract ethidium-positive dead cells to derive the total viable endothelial cell count. Corrected global cell density was calculated by dividing the number of viable cells by the graft area, which had been corrected for imaging a curved surface.

**Results:**

Corrected global cell density was lower than the central endothelial cell density in both groups: 85.5% of the pre-preparation central endothelial cell density in the peel group and 75.8% in the bubble group. Corrected global cell density was significantly lower in the liquid bubble separation group than in the peel group (p=0.04).

**Conclusions:**

Eye bank estimations of central endothelial cell density overestimate true cell density after graft preparation in DMEK. A peel method is less damaging and more consistent than a liquid bubble method. Cell loss correlated strongly with the degree of stromal hydration prior to bubble separation in the liquid bubble group.

## Introduction

Selective endothelial keratoplasty (EK) techniques have replaced penetrating keratoplasty (PK) as the leading treatment modality for corneal endothelial failure.^1^ Descemet membrane EK (DMEK), the latest iteration of EK, is reported to have better visual outcomes, faster visual recovery^2^ and lower rates of rejection^3^ than other forms of EK.

While data from single surgeon, high volume centres suggest that graft survival and endothelial cell (EC) loss in DMEK can approximate figures quoted for PK,^4^ data from large registries suggest that EK survival is significantly shorter than that for PK, with the worst results being reported for DMEK.^1^

Graft registry data have established a link between donor EC density (ECD) and graft survival.^5^ Extrapolation of PK survival data has been used to derive the minimum ECD required for an acceptable median graft survival time,^6^ with most eye banks adopting a minimum central ECD of approximately 2200 cells/mm^2^ as a cut-off for donor tissue use in transplantation.

Viable ECs do not cover the entire endothelial surface of any corneal transplant. Hypotonia-induced stromal folds are often devoid of cells, with the adjacent areas containing high numbers of dead and dying cells.^7^ ^8^ Additionally, iatrogenic endothelial damage occurs at every stage of the journey from donor retrieval to implantation, including storage,^9^ dissection,^10^ trephination,^11^ insertion^12^ or suturing.^13^ Consequently, patterns of cell damage vary between PK and EK. In EK, the insertion technique and the size of the wound used are also known to affect the patterns and degree of endothelial damage induced.^14^ This ‘transplantation trauma’ is thought to account for the significant drop in early postoperative ECD compared with donor ECD measured in the eye bank. Preoperative^9^ and early postoperative ECD has been shown to have a significant positive correlation with graft survival.^15^ Ongoing EC loss also occurs at a higher rate than in non-transplanted corneas.^6^

Given the relatively high rates of primary and early graft failure in DMEK,^1^ it is particularly important to minimise any tissue damage during donor tissue preparation. Preparation techniques described divide broadly into those based on peeling descemet membrane (DM) with forceps,^16^ ^17^ and techniques aiming to separate DM from the corneal stroma with a fluid injection^18–20^ (either air or liquid). Most evaluations of these techniques focus on macroscopic donor tissue integrity with little or no comment on endothelial viability. Where endothelial viability is evaluated, different techniques have been used, making direct comparisons difficult.

Evaluating EC viability in DMEK is more challenging than for other forms of EK, as DMEK donor tissue scrolls upon itself when immersed, and artefactual tissue trauma may occur when unrolling specimens for imaging.

The most commonly described method is based on dual staining with trypan blue and alizarin red.^12^ ^21^ There are several limitations to this approach. It is probable that trypan blue staining systematically underestimates EC mortality by failing to recognise apoptotic cells.^8^ Also, the trypan blue stained nuclei of individual dead cells may not be resolved by standard macroscopic photography.^22^ Smaller, non-contiguous areas of dead cells may therefore be included incorrectly in macroscopically viable areas. Extrapolation from small central high power fields to global estimates of viable ECD may also be problematic. In addition to the risk of sampling bias, ECD increases from the corneal apex to the periphery,^23^ and cell death is not uniform across the cornea.^7^ Methods of whole corneal assessment either ignore the biasing effect of corneal curvature or involve creating radial incisions to flatten the tissue.^8^ ^24^

Here, we describe an atraumatic method for imaging the entire donor specimen in DMEK at the cellular level, using nuclear staining to facilitate accurate automated cell counting and a calcein-AM/ethidium homodimer, live/dead assay, which may overcome some of the limitations listed above for trypan blue and alizarin red.^24^ Using this novel method for quantifying donor endothelial viability, we compare contemporary peeling and fluid bubble separation techniques for DMEK donor preparation.

## Methods

### Study tissue

The ethical approval for study protocol was received from the institutional review board. All procedures conformed to the tenets of the Declaration of Helsinki.

Human corneoscleral buttons with consent for research use were obtained from Miracles of Sight Eye bank, North Carolina, USA. Tissue processing and experimentation were performed by a single surgeon (MB) with experience of >100 DMEK tissue preparations. All tissue had a central EC count of >2200 cells/mm^2^ and had been stored in Optisol GS (Bausch & Lomb, Rochester, New York, USA) for no longer than 14 days prior to experimentation. Initial cell density measurement was performed by specular microscopy in the eye bank supplying the tissue. Prior to experimentation, specimens were assessed using 0.4% trypan blue (Sigma, St. Louis, Missouri, USA) and excluded if large areas of cell loss were visible.

Samples were assigned to one of two methods of DMEK preparation (n=8 in each group). When paired tissue was available, one cornea from each pair was assigned to each intervention. Unpaired tissue was assigned alternately.

### Interventions

Two alternate methods of DMEK preparation were compared: *manual peeling*,^16^ using a technique widely applied to prepare DMEK tissue immediately prior to surgery, and *liquid bubble separation*,^20^ following the method described by Europe's largest Eye Bank (the Venice Eye Bank).

#### Manual peeling

Corneoscleral rims were placed on a punch block from the Coronet trephine (Network Medical Products, Yorkshire, UK). Using a blunt Sinsky hook, the peripheral DM was scored circumferentially (360°) 0.5 mm anterior to Schwalbe’s line. An area of the scored DM edge free of radial tags was grasped with a single pair of fine non-toothed forceps and the DM peeled away from the underlying stroma approximately half way across the cornea under balanced salt solution (BSS). The DM was then replaced flat against the stroma by drying liquid away from beneath the peeled DM using a cellulose sponge, and a partial thickness 8.00 mm trephination performed. The redundant peripheral ring of endothelium between the trephination and score site was removed to confirm complete 360° trephination. The free edge of the 8.00 mm diameter DMEK donor specimen was then grasped at a single point, and donor peeling was completed under BSS.

#### Liquid bubble separation

Corneoscleral rims were placed on a punch block, as described above. A 25-gauge needle mounted on a 2.5 mL syringe filled with Optisol GS was inserted superficially through the tissue just peripheral to the pigmented trabecular meshwork and advanced until the entire bevel was located 0.5 mm anterior to Schwalbe's line. Optisol GS was injected in 0.1 mL aliquots until a peripheral separation ‘bubble’ between the stroma and DM could be seen. If 0.3 mL of liquid had been injected into the stroma without a successful bubble formation, the same process was attempted at a site 180° opposite. The total amount of liquid injected into the stroma prior to initial bubble formation was recorded for each specimen. Once a bubble had been initiated, liquid was injected until the bubble had spread to the trabecular meshwork at the opposite site of the corneal button. The peripheral DM was subsequently pierced with a 27-gauge needle and liquid was drawn from the bubble space using a cellulose sponge placed on the scleral rim. Once all visible liquid had been removed, an 8.00 mm trephination was performed to complete the preparation of the DMEK donor specimen.

### Specimen preparation

Prepared DMEK donor scrolls were left on the stromal surface of the corneoscleral button and placed into a 12-well plate. Each sample was covered with 250 μL of BSS containing Hoechst 33342 (10 μM), ethidium homodimer II(4 μM) and calcein-AM (2 μM) (Invitrogen, Carlsbad, California, USA) and incubated at 37°C for 30 min.

Hoechst 33342 and ethidium homodimer II are nuclear binding dyes that do not fluoresce until they are bound to nuclear DNA. Hoechst 33342 readily penetrates intact cell membranes and binds to the nuclear DNA of all ECs (both living and dead). Ethidium homodimer II is only able to penetrate compromised cell membranes of dead/dying (necrosis and late apoptosis). Calcein-AM readily travels through the cell membrane and is metabolised to brightly fluorescent calcein by cytosolic esterases. Dead cells are either unable to metabolise calcein-AM to fluorescent calcein or unable to retain fluorescent calcein owing to their compromised cell membranes. Hoechst, ethidium and calcein are visible in the DAPI, TRITC and FITC channels of the fluorescence microscope, respectively.

**Figure 1:**
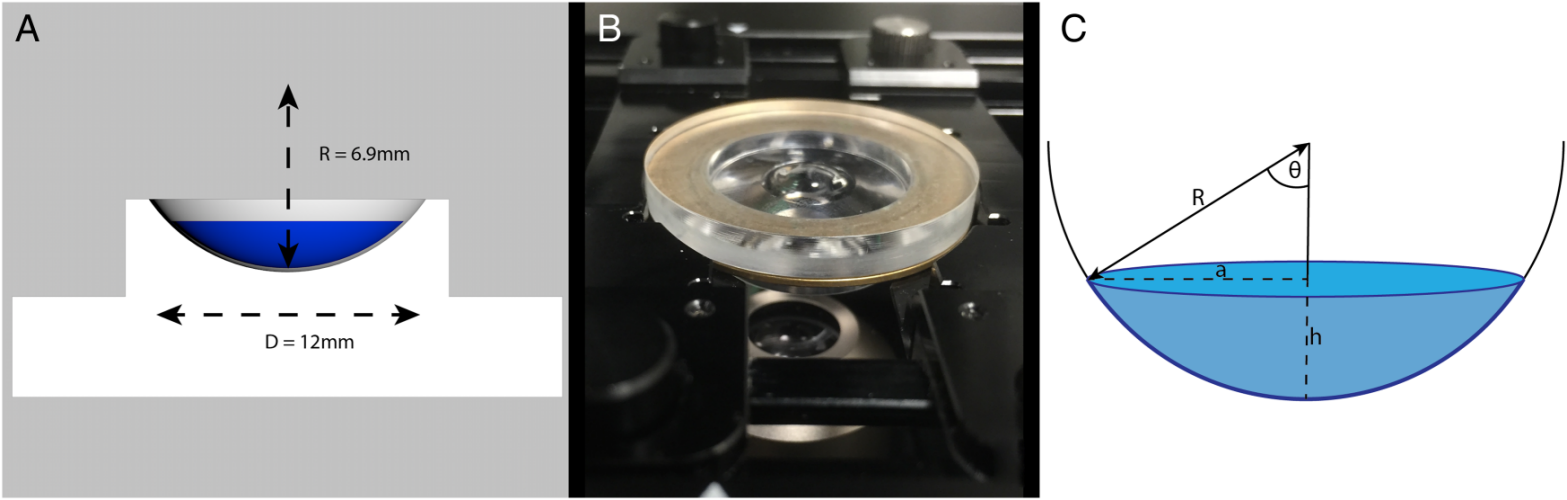
(A) Schematic diagram of the customised Descemet membrane endothelial keratoplasty (DMEK) imaging chamber. A radius of curvature of 6.9 mm was chosen to match the standard posterior corneal curvature, allowing DMEK donor specimens to lie flat, without wrinkles, in their native orientation. (B) Photograph of customised imaging chamber inverse mounted in the microscope stage for image acquisition. (C) The true area of the graft is that of a spherical cap and not a circle. Assuming that the graft is a circle underestimates the area and artificially increases the calculated cell density. True graft areas were calculated using the average measured graft diameter and the base curvature of the imaging chamber. The predicted area for a circle from an 8.00 mm trephine is 50.27 mm^2^. The area of a spherical cap is calculated as 2πRh, since

After incubation and staining, the samples were gently rinsed with BSS and mounted for imaging in a customised chamber designed to match the curvature of the DMEK donor (figure 1A). Drops of BSS and the capillary action of partial drying with absorbent cellulose sponges were used in a ‘no touch’ technique to orientate the DMEK donor samples flat on the curved surface, endothelial side up. The endothelial surface was then covered with viscoelastic (Provisc, Alcon, Fort Worth, Texas, USA) before chamber and imaging with the ×4 objective (Nikon CFI Plan Fluor 4X) of a Nikon Eclipse Ti-E inverted fluorescence microscope (Nikon, Tokyo, Japan; figure 1B). Dimensions from the curved imaging chamber were used to calculate the true graft area (figure 1C)

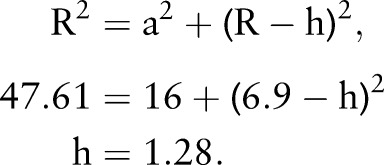

True area of 8.00 mm DMEK transplant=55.49 mm^2^ (2πRh=2π×6.9×1.28). Individual calculations for the transplants were made using average graft diameter.

To image the entire graft surface, multiple image tiles were combined and stitched together using the integrated software accompanying the microscope (NIS Elements AR V.4 (Nikon, Tokyo, Japan)). Each image tile consisted of a z-stack of 20–40 individual images. In-focus information from each image was combined using the enhanced depth of focus software module to produce a single tile. With this technique, it was possible to visualise every individual cell across the entire graft surface.

### Image processing and outcome measures

**Figure 2:**
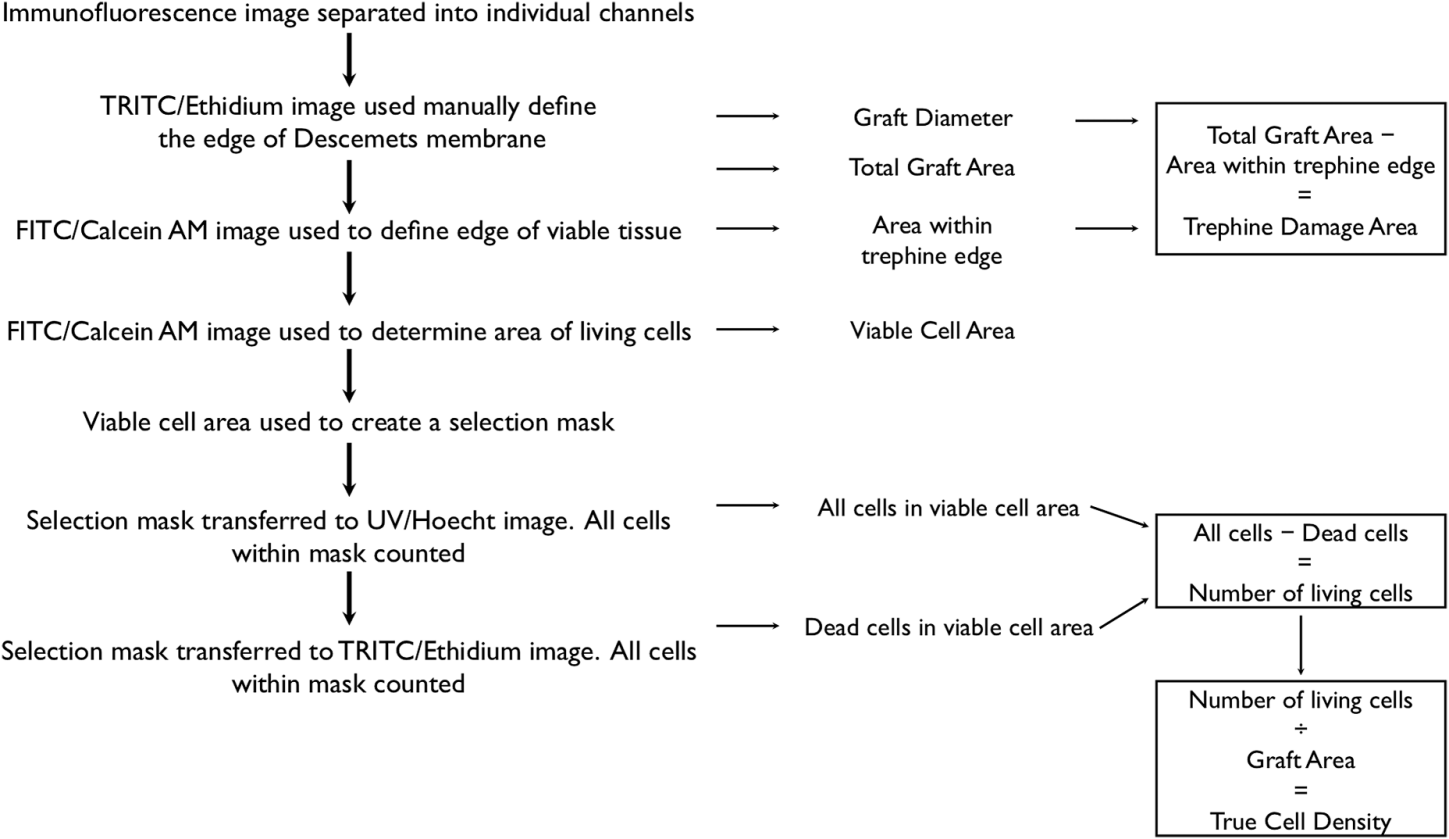
Flow chart outlining image processing and quantification steps.

**Figure 3:**
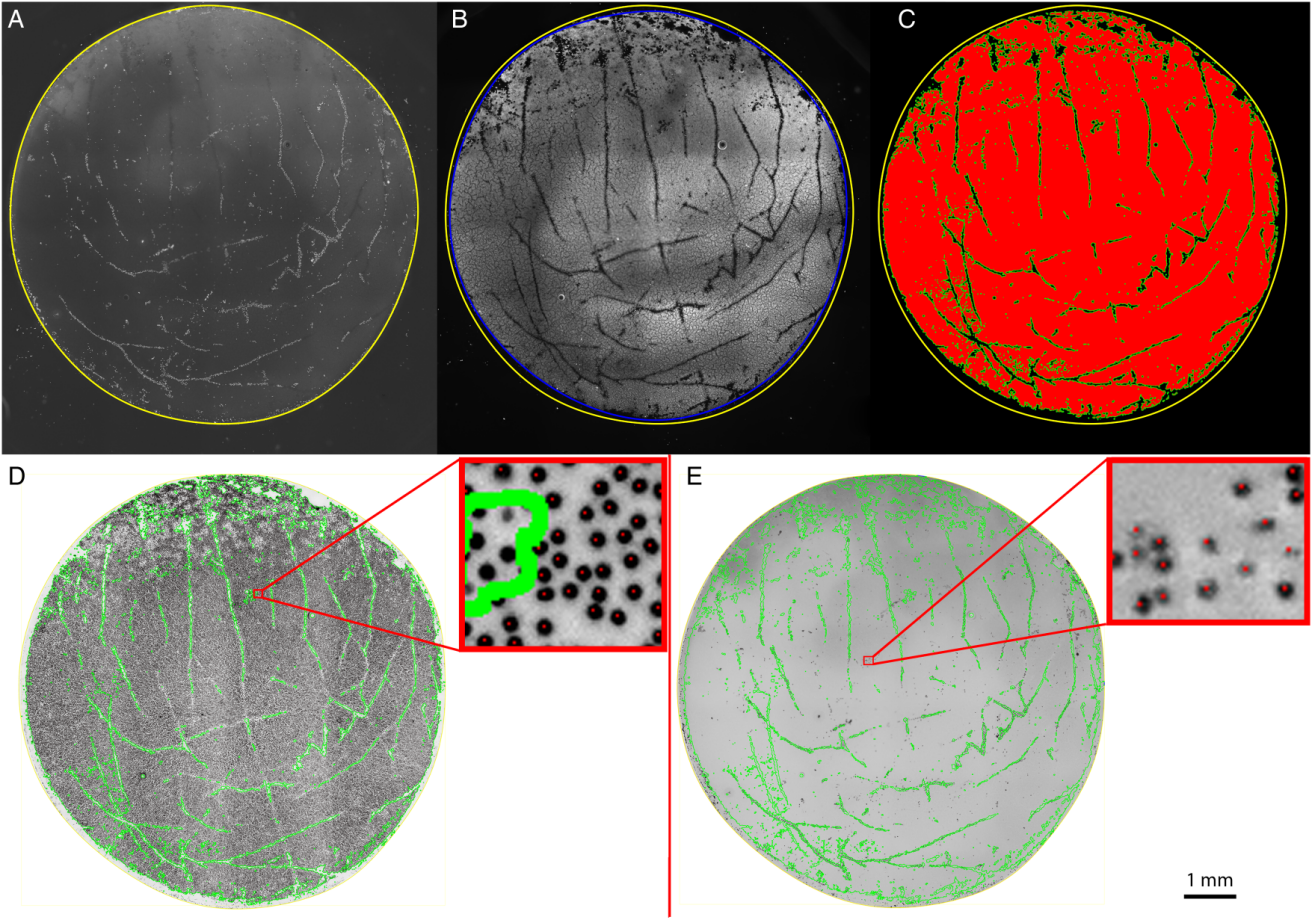
(A) TRITC/ethidium channel image showing manual demarcation of graft edge using the polygon selection toll in Image J (yellow outline). (B) Yellow line defined in the TRITC/ethidium image is copied and applied to the FITC/calcein channel image. The edge of calcein-positive area is defined in the same way as the graft edge (blue outline). The difference in the areas of the yellow and blue selections was defined as the trephination damage area. (C) The FITC/calcein channel image is thresholded and segmented. The area of living cells (red area) is used to create a selection mask (green lines). (D) Ultraviolet/Hoechst channel images were colour inverted and the viable graft area selection applied (green outline). Each nucleus (black dot) within the selection mask is counted. Zoomed in area (top right) showing all nuclei within the mask have been counted, as shown by the presence of a red dot in the centre of nucleus. Nuclei outside the mask are not counted (no red dot in centre of nucleus). (E) TRITC/ethidium channel image is inverted, and the living area selection is applied (green outline). Each nucleus (black dot) within the selection mask is counted. Zoomed in area (top right) showing ethidium-staining nuclei within the mask have been counted, as shown by the presence of a red dot in the centre of nucleus.

Images were exported to the open source NIH ImageJ software (National Institutes of Health, Bethesda, Maryland, USA) for processing and analysis (figure 2). For each DMEK specimen, the graft area and the area of viable cells were manually defined (figure 3A, B), and the differences between the two used to calculate the damage caused by tissue trephination.

The FITC/calcein channel image underwent a process of thresholding, segmentation and processing to remove image artefacts and background noise (figure 3C, red area). This area was used to create a mask using the ‘create selection’ function in ImageJ. From this binary image (figure 3C, green outline), we were able to calculate the percentage area of the graft covered by viable cells (=viable cell area/total graft area) and the percentage cell coverage excluding the trephination damage area (=viable cell area/total graft area—trephination damage area).

All ultraviolet/Hoechst and TRITC/ethidium-staining nuclei within the viable cell area were identified and counted using the ITCN nucleus counting plugin for ImageJ (figure 3D, E), after this method had been validated against manual counts.

The viable graft area included cells staining with both ethidium and calcein. These may represent dying cells within the endothelial monolayer or dead cells that have become detached and are sitting on top of the monolayer.

Total viable cell number was calculated by subtracting the number of ethidium-positive cells from the number of Hoechst-positive cells. Corrected global ECD was then calculated by dividing viable cell number by the corrected graft area. The corrected global ECD (total viable ECs/graft area) was normalised for each sample by dividing it by the measured central cell density taken from a central area of confluent cells.

### Analysis

Statistical comparisons were performed using GraphPad Prism (GraphPad Software, La Jolla, California, USA) using two-tailed t tests with a threshold for statistical significance set at p<0.05. A qualitative comparison of patterns of cell loss was also performed.

## Results

### Comparison of peeling versus liquid separation

There were no statistically significant differences in donor age, death to storage time or eye bank measured central ECD between the two groups (pre-preparation graft characteristics are presented in table 1).

**Table 1:** Baseline characteristics of graft tissue used in each experimental group

Measurement	Peel (n=8)	Liquid bubble (n=8)	p Value
Eye bank ECD (specular)	2790±290 cells/mm^2^	2878±317 cells/mm^2^	0.35
Donor age	57.1±12.8 years	53.4±16.9 years	0.62
Death to storage time	10.6±2.8 h	10.4±3.2 h	0.76
Time in Optisol GS	10.8±2.8 days	11.1±2.2 days	0.69

ECD, endothelial cell density.

The percentage area covered by viable cells was significantly higher in the peel group.

In spite of using the same diameter trephine in all samples, average graft area was significantly higher in the DM bubble group. There was no statistically significant difference in the area of damage caused by trephination between the two groups (table 2).

**Table 2:** Differences in graft area, viable cell area and trephine damage between grafts prepared using peeling versus liquid bubble separation

Measurement	Peel (n=8)	Liquid bubble (n=8)	p Value
Graft area—flat	49.9±0.51 mm^2^	52.6±0.73 mm^2^	<0.001
Graft area—spherical cap	55.0 mm^2^	58.3 mm^2^	
Maximum graft diameter	8.1±0.5 mm	8.5±0.7 mm	
Minimum graft diameter	7.7±0.4 mm	8.1±0.6 mm	
Trephine damage area	2.7±0.3 mm^2^	3.4±0.3 mm^2^	0.09
Percentage area covered by viable cells	87.7±1.4%	75.5±5.6%	0.04
Percentage area covered by viable cells within area of trephine damage	92.9±1.1%	81.0±6.3%	0.05

**Figure 4:**
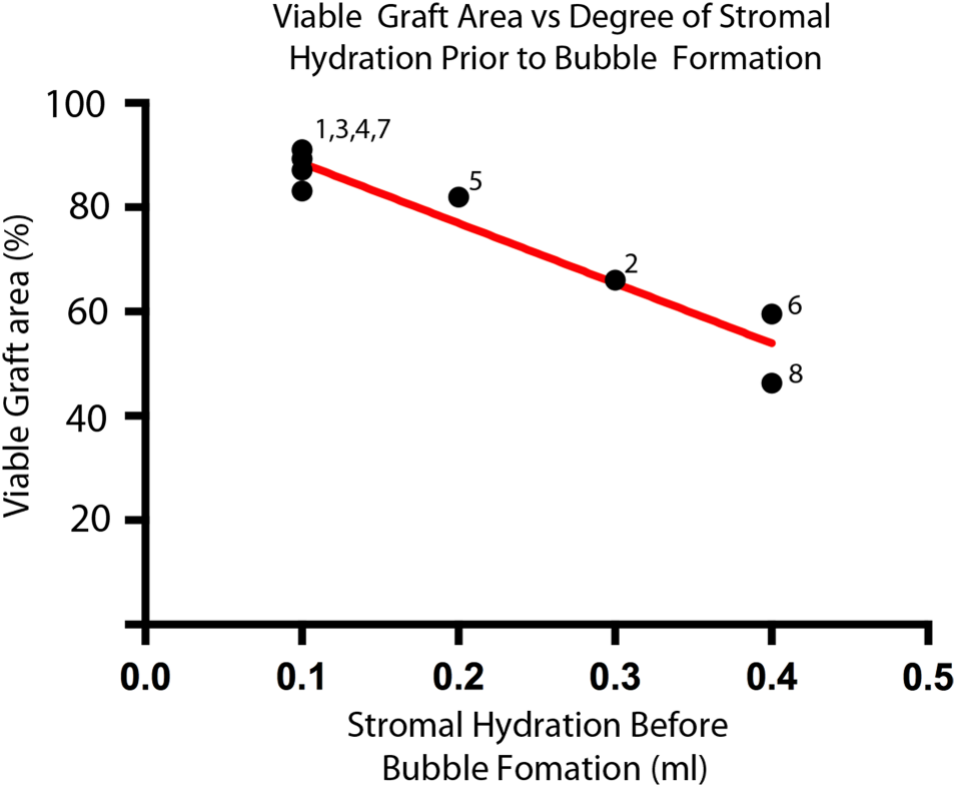
Graph of area covered by viable cells against the amount of fluid injected prior to bubble formation. The numbers indicate the order of each sample in the experiment and show that increased stromal hydration was not limited to the early cases.

After individual cell viability assessment, the total number of living cells was divided by the true graft area to give a global cell density. This was divided by the central ECD to correct for starting variations in cell density and expressed as a percentage of the central ECD. Corrected global cell density was 85.5±4.7% of central ECD in the peel group and 75.8±12.4% of central ECD in the bubble group, p=0.04. We observed a larger variation in tissue viability for the liquid bubble method of donor tissue preparation. To investigate this further, a post hoc comparison between the amount of liquid injected into the stroma prior to DM bubble formation (stromal hydration) and graft viability was made using laboratory notes from the experimentation. There was a strong correlation between the amount of tissue hydration and the area of viable cells (R=−0.96, p<0.001; figure 4).

### Patterns of cell loss

**Figure 5:**
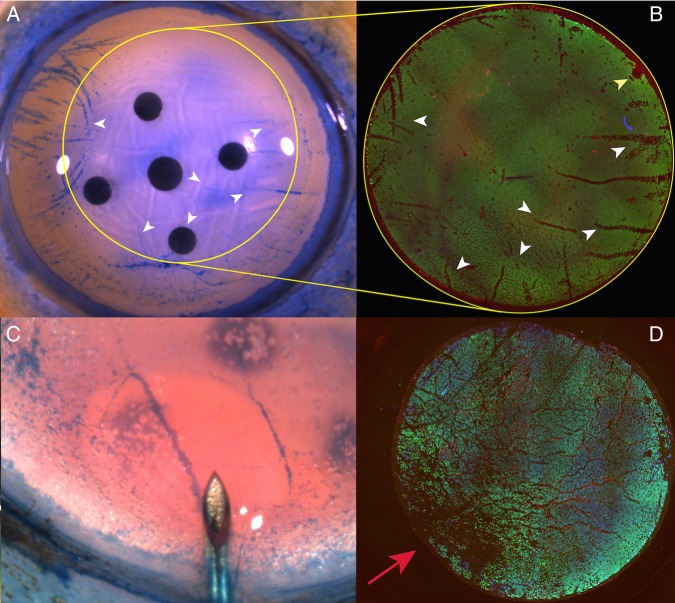
(A) Photomicrograph showing areas of cell damage staining with trypan blue on the corneoscleral button prior to peeling and trephination (white arrow heads). Yellow outline shows the site of trephination, which was off-centred to avoid the site of a previous corneal incision for cataract surgery. Yellow outlines shows site of trephination. (B) Immunofluorescence image of peeled Descemet membrane endothelial keratoplasty, areas of non-viable cells within the trephination area closely match those observed on the button prior to peeling but are easier to identify using this method (white arrow heads). Site of forceps fixation is marked with a yellow arrowhead. (C) Initial stromal hydration occurs prior to a peripheral Descemet membrane fluid bubble occurring. (D) The site of stromal hydration corresponds with maximal endothelial damage (red arrow) and cannot always be avoided by off-centred trephination.

Linear areas of cell loss corresponding to folds in the tissue could be seen in both groups and closely corresponded to the observed pattern witnessed using trypan blue staining prior to tissue preparation (figure 5A, B). In the DM bubble group, an area of increased cell loss was often observed at the point of initial bubble formation (figure 5C, D).

## Discussion

We have described a novel method of DMEK graft viability assessment that eliminates the need for radial incisions to flatten graft tissue, limits iatrogenic mounting trauma and fully accounts for variations in cell density and cell death across the whole specimen. To our knowledge, this represents the first method of endothelial viability assessment conducted on an individual cell level across the entire surface of a corneal transplant in living tissue.

Using this technique, we found that a peeling method, commonly used by surgeons preparing grafts in theatre and employed by eye banks shipping pre-stripped tissue in the USA (Miracles of Sight Eye Bank) and Europe (Amnitrans Eye Bank, Rottterdam, the Netherlands), is reliable and produces minimal EC damage. The percentage area of non-viable tissue in the peeled samples was 12%, however, 5% of the non-viable graft area is attributable to trephination damage alone. Qualitative comparison with trypan blue staining prior to tissue dissection suggests that the majority of non-viable areas are present on the transplant tissue prior to preparation, with non-viable cells concentrated within tissue folds.

The corneal endothelium heals primarily by a process of wound repair in which cells enlarge and spread with minimal cell division occurring in vivo.^25^ Cell loss during tissue storage and preparation is therefore likely to map directly to postoperative ECD^9^ and the prognosis for DMEK graft survival. A detailed understanding of where cell loss can be avoided should ultimately improve graft outcomes and survival. We were able to detect a significant difference between two DMEK preparation techniques, where other authors using more simplistic methods could not.^26^ Although our method is more time-consuming, we believe it significantly improves upon those previously described assessments in whole grafts.^27^ ^28^

Our findings compare favourably with those reported by other authors assessing endothelial viability in DMEK preparation. Jardine *et al*^27^ used calcein-AM alone to assess areas of cell loss after preparation of DMEK transplants using a peel technique. They found that 22.5% of the graft area was non-viable, and inspection of the figures in their paper suggests this may not include the damage caused by trephination. As acknowledged by the authors themselves, it is likely that the higher degree of cell damage reported previously is, in part, due to the method of tissue mounting used. Using our technique, we were able to minimise trauma associated with specimen manipulation. Our relatively good results may therefore reflect the degree of cell damage associated with DMEK donor preparation more accurately.

The amount of stromal hydration accounts for approximately 85% (R^2^) of the variability in endothelial damage observed in the liquid separation group. Excessive stromal hydration increases tissue strain,^22^ and this is likely to transfer mechanical stress directly to the ECs. Tissue distortion and mechanical stress have been implicated in both acute EC trauma^7^ and cell death during tissue storage.^20^ In grafts in which a separation plane under the DM could be induced with minimal hydration, tissue viability was comparable with that of a peel technique. We did not observe a learning effect whereby excessive tissue hydration was limited to the earlier samples in the liquid bubble group. Instead, the amount of liquid needed may relate to the adhesion between the stroma and DM and be an intrinsic property of the tissue itself. It has been suggested that grafts stored in organ culture without dextran may be easier to separate using a liquid separation method^20^ and therefore induce less endothelial damage. Direct transfer of methods of DMEK preparation tested in organ culture to corneas in cold storage may not be appropriate and specific validation should be carried out for each storage method.

In addition to counting all viable cells, our method also takes into account the true surface area of the curved posterior corneal surface when calculating cell density. Since the area of a spherical cap is greater than that of a circle of the same diameter, previous studies may have systematically overestimated cell density at the point of transplantation.^29^ Global cell density was on average 14.6% lower than central cell density in the peel group and 24.2% lower in the bubble group, suggesting that an early postoperative ECD reduction of up to 25% could be expected from tissue preparation alone.

For the preparation of DMEK scrolls from cold stored corneal tissue, based on results here, we would recommend techniques based on peeling rather than fluid bubble separation. Higher cell damage for the liquid bubble technique observed here may not translate to techniques for deep anterior lamellar keratoplasty, since corneal ECs undergo changes in storage that make them less resistant to mechanical damage.^30^

A future simple method of reducing endothelial mortality may involve limiting the damage induced by trephination. Further experimentation to determine whether ongoing cell loss during storage of pre-prepared DMEK grafts differs between the peel and bubble separation methods is warranted.

## References

[1] Coster DJ, Lowe MT, Keane MC, et al A comparison of lamellar and penetrating keratoplasty outcomes: a registry study. Ophthalmology 2014;121:979–87. 10.1016/j.ophtha.2013.12.017 24491643

[2] Guerra FP, Anshu A, Price MO, et al Endothelial keratoplasty: fellow eyes comparison of Descemet stripping automated endothelial keratoplasty and Descemet membrane endothelial keratoplasty. Cornea 2011; 30:1382–6. 10.1097/ICO.0b013e31821ddd25 21993468

[3] Anshu A, Price MO, Price FW Risk of corneal transplant rejection significantly reduced with Descemet's membrane endothelial keratoplasty. Ophthalmology 2012;119:536–40. 10.1016/j.ophtha.2011.09.019 22218143

[4] Feng MT, Price MO, Miller JM, et al Air reinjection and endothelial cell density in Descemet membrane endothelial keratoplasty: five-year follow-up. J Cataract Refract Surg 2014;40:1116–21. 10.1016/j.jcrs.2014.04.023 24957432

[5] Bourne WM Cellular changes in transplanted human corneas. Cornea 2001;20:560–69. 10.1167/iovs.02-1255 11473153

[6] Armitage WJ, Dick AD, Bourne WM Predicting endothelial cell loss and long-term corneal graft survival. Invest Ophthalmol Vis Sci 2003;44:3326–31. 10.1167/iovs.02-1255 12882777

[7] Albon J, Tullo AB, Aktar S, et al Apoptosis in the endothelium of human corneas for transplantation. Invest Ophthalmol Vis Sci 2000;41: 2887–93. 10967041

[8] Gain P, Thuret G, Chiquet C, et al Value of two mortality assessment techniques for organ cultured corneal endothelium: trypan blue versus TUNEL technique. Br J Ophthalmol 2002;86:306–10. 10.1136/bjo.86.3.306 11864889 PMC1771045

[9] Thuret G, Chiquet C, Bernal F, et al Prospective, randomized clinical and endothelial evaluation of 2 storage times for corneal donor tissue in organ culture at 31°C. Arch Ophthalmol 2003;121:442–9. 10.1001/archopht.121.4.442 12695240

[10] Suwan-Apichon O, Reyes JMG, Griffin NB, et al Microkeratome preparation of lamellar corneal grafts. Eye Contact Lens 2006;32:248–9. 10.1097/01.icl.0000219364.21999.e4 16974159

[11] Terry MA, Saad HA, Shamie N, et al Peripheral endothelial cell damage after trephination of donor tissue. Cornea 2009;28:1149–52. 10.1097/ICO.0b013e3181a87a28 19770708

[12] Saad HA, Terry MA, Shamie N, et al An easy and inexpensive method for quantitative analysis of endothelial damage by using vital dye staining and Adobe Photoshop software. Cornea 2008;27:818–24. 10.1097/ICO.0b013e3181705ca2 18650669

[13] Alqudah AA, Terry MA, Straiko MD, et al Immediate endothelial cell loss after penetrating keratoplasty. Cornea 2013;32:1587–90. 10.1097/ICO.0b013e3182a73822 24145632

[14] Terry MA, Saad HA, Shamie N, et al Endothelial keratoplasty: the influence of insertion techniques and incision size on donor endothelial survival. Cornea 2009;28:24–31. 10.1097/ICO.0b013e318182a4d3 19092400

[15] Nishimura JK, Hodge DO, Bourne WM Initial endothelial cell density and chronic endothelial cell loss rate in corneal transplants with late endothelial failure. Ophthalmology 1999;106:1962–5. 10.1016/S0161-6420(99)90409-8 10519593

[16] Tenkman LR, MD FWPJ, Price MO Descemet membrane endothelial keratoplasty donor preparation: navigating challenges and improving efficiency. Cornea 2014;33:319–25. 10.1097/ICO.0000000000000045 24452215

[17] Yoeruek E, Bartz-Schmidt KU, Schmidt B Novel surgical instruments facilitating Descemet membrane dissection. Cornea 2013;32:523–6. 10.1097/ICO.0b013e3182588ae9 22902493

[18] Muraine M, Gueudry J, He Z, et al Novel technique for the preparation of corneal grafts for Descemet membrane endothelial keratoplasty. Am J Ophthalmol 2013;156:851–9. 10.1016/j.ajo.2013.05.041 23932263

[19] Busin M, Scorcia V, Patel AK, et al Donor tissue preparation for Descemet membrane endothelial keratoplasty. Br J Ophthalmol 2011;95:1172–3; author reply 1173 10.1136/bjo.2010.195651 21169271

[20] Parekh M, Ruzza A, Salvalaio G, et al Descemet Membrane Endothelial Keratoplasty tissue preparation from donor corneas using a standardized submerged hydro-separation method. Am J Ophthalmol 2014;158:277–285.e1. 10.1016/j.ajo.2014.04.009 24792104

[21] Spence DJ, Peyman GA A new technique for the vital staining of the corneal endothelium. Invest Ophthalmol 1976;15:1000–2. 62728

[22] Sperling S Early morphological changes in organ cultured human corneal endothelium. Acta Ophthalmol 1978;56:785–92. 10.1111/j.1755-3768.1978.tb06643.x 80914

[23] Amann J, Holley GP, Lee S-B, et al Increased endothelial cell density in the paracentral and peripheral regions of the human cornea. Am J Ophthalmol 2003;135:584–90. 10.1016/S0002-9394(02)02237-7 12719063

[24] Pipparelli A, Thuret G, Toubeau D, et al Pan-corneal endothelial viability assessment: application to endothelial grafts predissected by eye banks. Invest Ophthalmol Vis Sci 2011;52:6018–25. 10.1167/iovs.10-6641 21666243

[25] Doughman DJ Prolonged donor cornea preservation in organ culture: long-term clinical evaluation. Trans Am Ophthalmol Soc 1980;78:567–628. 7020215 PMC1312154

[26] Yoeruek E, Bayyoud T, Hofmann J, et al Comparison of pneumatic dissection and forceps dissection in Descemet membrane endothelial keratoplasty: histological and ultrastructural findings. Cornea 2012;31:920–5. 10.1097/ICO.0b013e31823f7870 22511023

[27] Jardine GJ, Holiman JD, Stoeger CG, et al Imaging and quantification of endothelial cell loss in eye bank prepared DMEK grafts using trainable segmentation software. Curr Eye Res 2014;39:894–901. 10.3109/02713683.2014.887120 24588207

[28] Kobayashi A, Murata N, Yokogawa H, et al Evaluation of internationally shipped prestripped donor tissue for Descemet membrane endothelial keratoplasty by vital dye staining. Cornea 2015;34:225–7. 10.1097/ICO.0000000000000330 25522223

[29] Lie JT, Birbal R, Ham L, et al Donor tissue preparation for Descemet membrane endothelial keratoplasty. J Cataract Refract Surg 2008;34:1578–83. 10.1016/j.jcrs.2008.05.036 18721723

[30] Koh S-WM, Gloria D, Molloy J Corneal endothelial autocrine VIP enhances its integrity in stored human donor corneoscleral explant. Invest Ophthalmol Vis Sci 2011;52:5632–40. 10.1167/iovs.10-5983 21482640 PMC3176069

